# Mu rhythm motor–auditory delay in imagined speech mirrors overt speech timing

**DOI:** 10.1038/s41598-026-37421-1

**Published:** 2026-01-28

**Authors:** Francesco Mantegna, David Poeppel, Joan Orpella

**Affiliations:** 1 Present Address: Department of Psychology, New York University, New York, NY 10003 USA; 2 Present Address: Department of Engineering Science, Oxford University, Oxford, Oxfordshire UK; 3 Present Address: Department of Neuroscience, Georgetown University Medical Center, Washington, DC 20007 USA

**Keywords:** Covert speech, Sensorimotor coordination, Efference, Magnetoencephalography, Event-Related Desynchronization, Neuroscience, Psychology, Psychology

## Abstract

Speaking—whether overtly or covertly—requires a mapping between motor commands and their sensory consequences, a process of sensorimotor coordination. The timing of sensorimotor coordination during overt speech is relatively well established. Here we asked whether during imagined speech sensorimotor coordination can preserve this timing and remain grounded in the same biophysical constraints underlying vocal articulation. We instructed participants to imagine producing visually presented syllables (/pa/, /ta/, /ka/). Using magnetoencephalography (MEG), we investigated the spatiotemporal dynamics of mu rhythm (8–30 Hz) power suppression. Cluster-based permutation analysis reveals a segregation of alpha (8–12 Hz) and beta (15–30 Hz) frequencies to auditory and motor areas, respectively. Latency analyses show that beta suppression in motor areas precedes alpha suppression in auditory areas by ~ 120 ms. This delay closely matches sensorimotor coordination time windows previously reported for overt speech. While prior work provided only indirect evidence for the temporal equivalence between imagined and overt speech—by probing the system with altered auditory feedback—our findings offer direct evidence by measuring strictly internal neural processes. Together, the results demonstrate the suitability of alpha–beta power suppression as a neural marker that separately indexes motor and auditory processes associated with imagined speech production.

**Supplementary Information:**

The online version contains supplementary material available at 10.1038/s41598-026-37421-1.

## Introduction

Skilled motor control relies on the precise coordination of perception and action^1^. To achieve desired outcomes, an agent must understand how actions map onto their sensory consequences. In speech, two complementary mappings relate articulatory movements to different types of sensory feedback: auditory feedback (the sounds we produce) and somatosensory feedback (the tactile and proprioceptive sensations arising from movements of the vocal tract). For example, an opera singer continually calibrates diaphragm contraction, airflow, and vocal tract configuration against an expectation of the target sound. Yet perception and action rely on fundamentally different physical processes, biological systems, and neural representations: movements arise from muscle contractions, whereas sounds are perceived as vibrations transmitted through air. Linking these domains requires a mapping between motor-output and sensory-input coordinate systems. Such linking operations typically incur processing delays, reflecting the computational cost of translating between distinct representational formats^2,3^.

Here, we investigate the timing of sensorimotor coordination during speech imagery—a condition in which no movement is executed, and no sensory consequences are perceived^4^. Our aim is to test whether the timing of imagined speech matches that of overt speech, achieving temporal equivalence. Temporal equivalence between imagery and overt speech may only emerge under certain conditions, namely when the mental simulation closely mirrors the physical act of speaking. In such cases, imagery can act as a faithful neural reenactment that preserves the temporal scaffolding of speaking^5,6^, rather than as a temporally compressed mental simulation that is unconstrained by the biomechanics of articulation^7,8^. To recreate these conditions, we designed our experiment around consonant–vowel syllables (/pa/, /ta/, /ka/), which emphasize low-level acoustic and articulatory features and minimize higher-order linguistic processing. This approach maximizes the likelihood of capturing timing patterns in imagery that reflect the same biophysical constraints governing overt speech.

To assess temporal equivalence, we need an estimate of the duration of sensorimotor coordination during overt speech that can serve as a reference to evaluate the timing of the corresponding processes during imagined speech. Previous estimates of this time window come from studies using auditory feedback perturbation paradigm^9^, in which the perceived auditory consequences of speech are artificially altered following articulation. Perturbations are often implemented as shifts in formant frequencies to selectively modify specific speech features. For instance, shifting the fundamental frequency (F0) can cause the speaker to perceive a different voice, while altering the first formant (F1) can lead to hearing a different phoneme (e.g., /a/ instead of /e/). These manipulations create mismatches between expected and perceived outcomes, measurable both behaviorally (e.g., compensatory responses) and neurally (e.g., mismatch responses). When perturbations are applied at different delays after speech onset, their effects disappear after roughly ~ 100 ms, suggesting that mismatches between motor and auditory consequences are normally integrated within this time frame^10,11^.

While some evidence suggests that the same temporal window for sensorimotor coordination may apply to imagined speech, prior studies have relied on external auditory probes, making their conclusions indirect^12^. Our goal here was to assess this timing more directly by focusing exclusively on neural activity generated by the imagery process itself, without any stimulation or feedback manipulation. This required a neural measure that could separately track motor and auditory components of speech imagery and do so with high temporal precision. Mu-rhythm power suppression—an oscillatory pattern which we here define as spanning the conventional alpha (8–12 Hz) and beta (15–30 Hz) bands—fits these requirements^13,14^. Previous work shows that beta suppression tends to localize to motor regions and alpha suppression to sensory regions^15^, and that mu suppression accompanies both overt and imagined movements across a variety of effectors^16,17^, including speech articulators^18^. Building on this, we adopted a two-step approach: first, identify whether motor-related beta and auditory-related alpha power suppression can be dissociated during speech imagery; and second, use the timing difference between these components as a direct index of sensorimotor coordination.

We used magnetoencephalography (MEG) to measure the temporal delay between motor- and auditory-related mu power suppression during imagined speech, interpreting this delay as a proxy for sensorimotor coordination. Participants imagined speaking visually presented syllables (/pa/, /ta/, or /ka/). We investigated mu-rhythm power suppression in both the alpha and beta bands using a cluster-based permutation test which revealed two distinct time–frequency clusters. Beta power suppression occurred earlier in motor regions, followed by alpha power suppression in auditory regions. Latency analyses showed that beta suppression consistently preceded alpha suppression by ~ 120 ms. This delay closely matches the sensorimotor coordination window reported for overt speech, suggesting that imagined speech preserves the same temporal scaffolding as overt speech. To further test this interpretation, we collected overt speech latencies in a behavioral pretest. Remarkably, the timing of both beta and alpha power suppression during imagery closely matched each participant’s own speech production latencies measured in this overt speech task, demonstrating that these neural signatures reflect the internally generated production of movement and sound. Together, these findings show that the temporal delay between motor beta and auditory alpha power suppression provides a direct, stimulation-free, neural measure of sensorimotor coordination during imagined speech.

## Results

### Speech onset distribution in the overt speech behavioral pretest

**Fig. 1:**
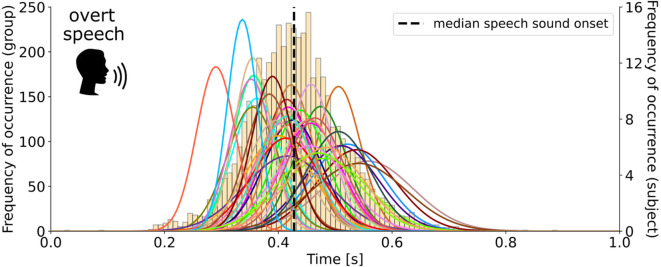
**Speech sound onsets measured in the overt speech behavioral pre-test**. Gaussian fits of the speech sound onset distribution across trials for each participant (colored lines) and a histogram of speech sound onsets across participants are represented. The dashed vertical line marks the median speech sound onset across participants.

Imagined speech lacks behavioral output that could serve as a reference for interpreting its underlying neural dynamics. Because no such measure can be obtained directly for imagined speech, we conducted an overt speech behavioral pretest to collect individual participant estimates of speech production latency. The distribution of speech sound onsets is shown in Fig. 1. Across participants, the median onset following visual stimulus presentation was 412 ms, representing the typical time required to initiate speech. Here, we define the speech sound onset as the release burst associated with the consonant vocalization. Because the target syllables (/pa/, /ta/, /ka/) begin with unvoiced stop consonants, the articulators start moving slightly before this acoustic event without producing any audible sound. Thus, the median onset provides a temporal anchor: just before this point, motor activity related to articulation should already be present, and just after this point, auditory activity should emerge in response to the produced sound. A Gaussian fit to the group-level onset distribution showed that latencies clustered tightly around the median, with a 25–75% interquartile range of 74 ms capturing variability across participants. Fitting each participant’s distribution individually revealed within-subject interquartile ranges from 54 ms to 157 ms, indicating that some speakers were more temporally consistent than others. Overall, the relatively narrow spread confirms that, as instructed, participants maintained consistent speech production latencies across trials. These overt speech onset measures—at both the individual and group level—will be used as temporal references for the following neural analyses.

### Comparison of MEG/EMG recordings during imagined and overt speech

**Fig. 2:**
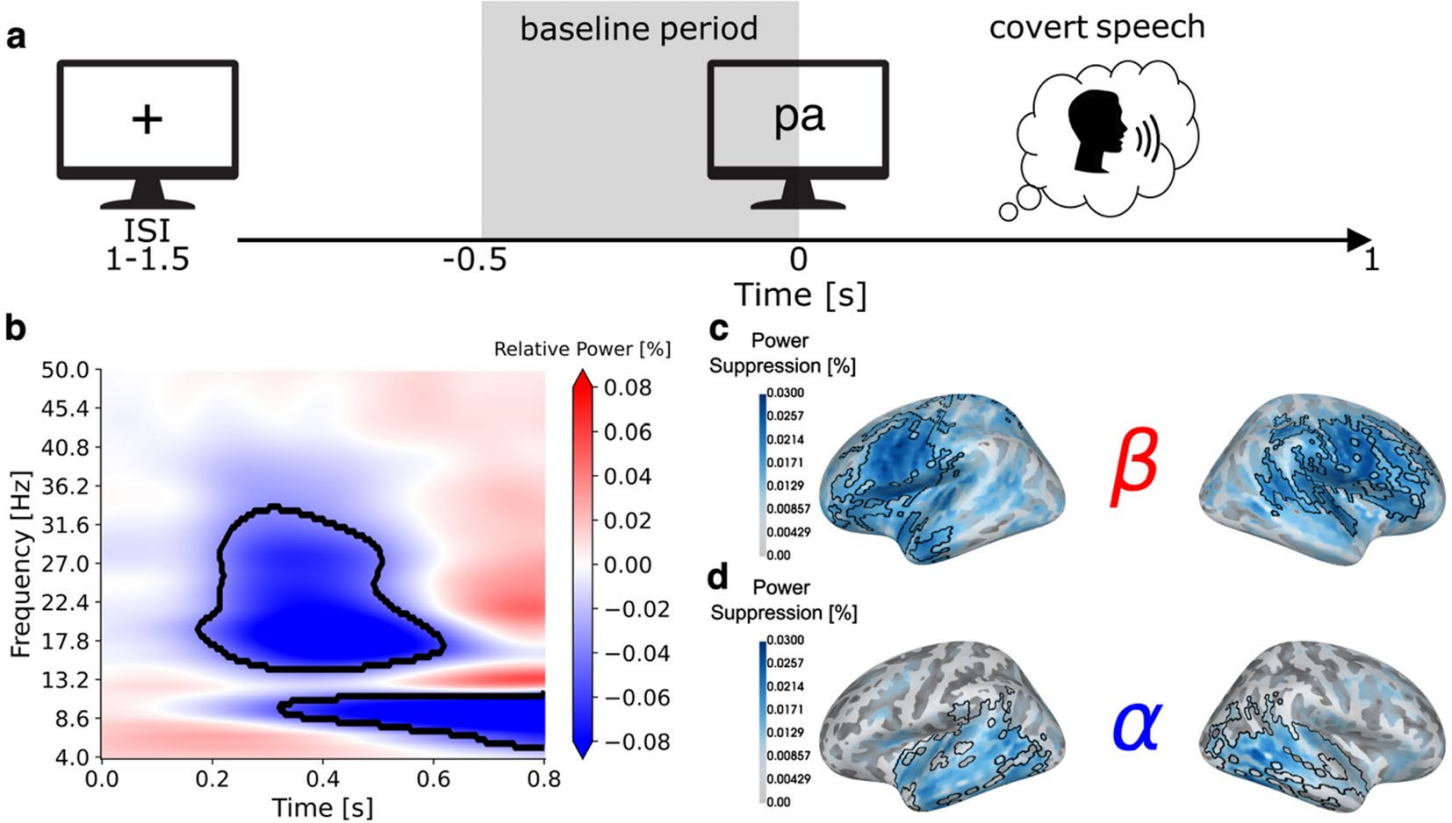
**Alpha and beta spectrotemporal clusters and their corresponding source localizations**. (**a**) A schematic representation of the experimental paradigm. (**b**) Sensor space time-frequency representation of non-phase-locked spectrotemporal modulations. Red and blue colors represent percentage increase and decrease in power with respect to the baseline period. The black contours represent the time-frequency regions that are consistent across participants as obtained from the cluster-based permutation test. Source localization of the beta (**c**) and alpha (**d**) power decrease clusters. The black contours represent the cortical vertices that are consistent across participants as obtained from the cluster-based permutation test.

In this study, participants were instructed to imagine articulating syllables (/pa/, /ta/, /ka/) presented visually in orthographic form (Fig. 2a). Task-elicited evoked responses showed a posterior to anterior progression across sensors, plausibly reflecting the sequential recruitment of visual, motor, and auditory areas (Fig. S1). One practical advantage of imagined speech is that it engages strictly internal neural processes: there is no overt movement execution, no self-produced sound, and no electromagnetic artifacts generated by speaking. As a proof of concept, we directly compared overt and imagined versions of the task for one representative participant. The results illustrate that overt articulation produces artifacts in the broadband signal (Fig. S2e) and pronounced distortions in the time–frequency representations (Fig. S2g), particularly in the lower and frontal MEG sensors closest to the participant’s mouth (Fig. S2h). In contrast, performing the same task covertly produces no observable artifacts. To ensure that participants were not moving during imagined speech, we recorded electromyography (EMG) from jaw and lip electrodes. In both overt and imagined speech, we observed EMG deflections of different magnitudes (Fig. S2b, f). Specifically, the EMG signal during overt speech was roughly ten times greater than during imagined speech. In line with previous studies, we interpret these small residual deflections in the imagined speech condition as micromovements^19^—minor, involuntary muscle activations that arise from incomplete inhibition of the motor command. Because overt speech neural recordings are heavily contaminated by artifacts and confounded by external sensory and motor aspects, we do not use them as a direct comparison in this study. Instead, we use overt speech production latencies from the behavioral pretest to guide interpretation of the imagined speech neural responses. The earliest prominent deflection in the grand-average EMG evoked response measured during imagined speech—corresponding to micromovement onset—occurred at approximately 250 ms, providing an additional temporal anchor for the neural responses (Fig. S3). Although single-trial and single-participant EMG signals were too unreliable and micromovements could only be characterized in aggregate format, this timing is still informative because it closely matches the articulatory-to-acoustic onset interval reported in previous overt speech studies^20^.

### Distinguishing induced vs. evoked frequency modulations

Next, we investigated the frequency modulations associated with imagined speech. In this study, the instruction to imagine speech was necessarily delivered via a visual cue, which inevitably triggers stimulus-driven neural responses. However, our interest is not in these externally evoked processes but in the internally generated processes associated with imagined speech in the absence of overt articulation. Therefore, the first step was to establish that the signals we are analyzing reflect self-generated rather than stimulus-driven activity. To do so, we ran a time–frequency analysis in sensor space and measured relative power change with respect to a pre-stimulus baseline (–0.5–0 s). The resulting time–frequency representations revealed a classic power decrease (event-related de-synchronization, ERD) in the mu-rhythm (8–30 Hz) centered around the median speech onset latency measured in the overt-speech behavioral pretest, suggesting that the suppression is linked to the imagined speech rather than being a simple response to the visual cue. To further test this interpretation, we conducted an analysis to assess the phase-locking of time-frequency responses across trials (Fig. S4a-b). This showed that mu-band frequency components persisted after subtraction of the evoked response, confirming that they are non-phase-locked, internally driven oscillations—unlike lower frequency components, which were phase-locked, stimulus driven oscillations.

### Spatial segregation of alpha-beta power suppression

Then, we asked whether the alpha (8–12 Hz) and beta (15–30 Hz) frequencies within the mu band serve different functional roles. To address this, we first ran a two-dimensional cluster-based permutation test (one-tailed, one-sample) on the time–frequency representations to identify spectrotemporal patterns showing significant power decrease relative to baseline across participants. Two distinct clusters emerged (Fig. 2b): a beta cluster (15–30 Hz) spanning ~ 0.25–0.55 s (*p* < 0.001) and an alpha cluster (8–12 Hz) spanning ~ 0.40–0.80 s (*p* < 0.001), already suggesting functional differentiation. Then, we examined their spatial localization. For each cluster, we projected absolute power during its time window and during the baseline into source space, we computed the relative power change, and we ran a cluster-based permutation test over cortical vertices to identify brain regions showing a power decrease relative to baseline across participants. The beta suppression localized to frontal motor regions (*p* < 0.005) and was absent in temporal areas (Fig. 2c), whereas the alpha suppression localized to temporal auditory regions (*p* < 0.01) and was absent in frontal motor areas (Fig. 2d). Together, these results reveal a frequency-specific, spatially segregated organization within the mu rhythm, indicating distinct functional roles for motor beta and auditory alpha.

### Temporal segregation between alpha and beta suppression

**Fig. 3:**
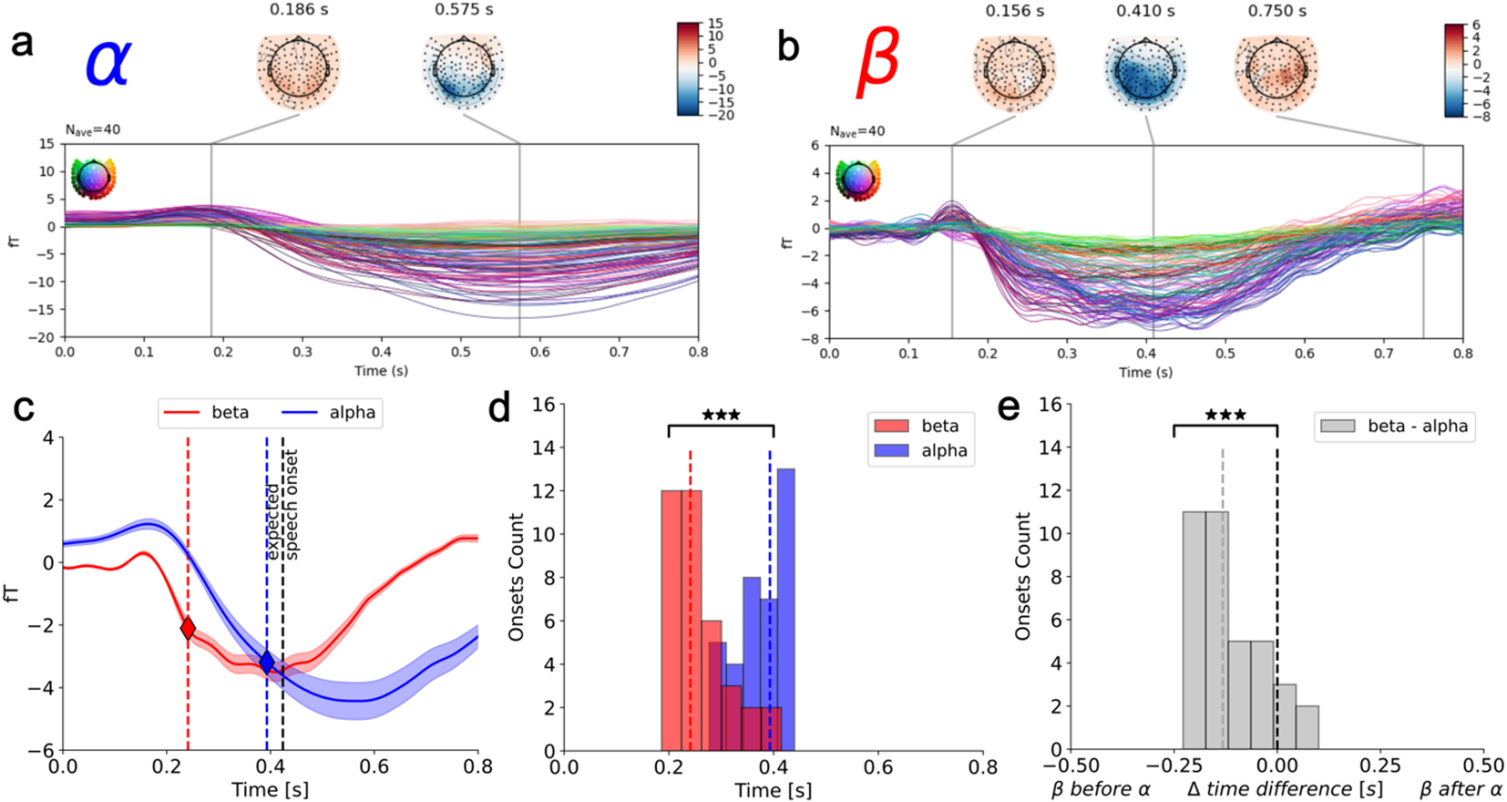
**Sensor space power decrease time course in the beta and alpha frequency bands**. Grand average Hilbert transformed band-filtered power decrease with respect to the baseline in the alpha (**a**) and beta (**b**) frequency bands. The lines represent the 157 axial gradiometers. MEG sensors are color-coded according to their spatial location on the scalp that is shown in the topography in the upper left. The topographies of most salient events in the time course are shown on top together with their timing. (**c**) The average across sensors of the baseline normalized envelope in the beta (red) and alpha (blue) frequency band is represented. The solid lines represent the mean and the shaded area surrounding the solid lines represent the standard error of the mean. The vertical dashed black line represents the expected speech onset measured in the behavioral pretest. The colored stars and vertical dashed lines represent the group-level beta (red) and alpha (blue) ERD onsets. (**d**) The distribution of individual subjects’ beta and alpha ERD onsets are represented in the red and blue histograms, respectively. The colored vertical dashed lines represent the median beta and alpha ERD onsets across participants. (**e**) The distribution of individual subjects’ temporal difference between beta and alpha ERD onsets is represented in the grey histogram. The black vertical dashed line represents no temporal difference; the grey vertical dashed line represents the median temporal difference between beta and alpha ERD onsets. A negative value indicates that the beta onset precedes the alpha onset, a positive value indicates that the beta onset follows the alpha onset.

We next investigated whether there is a temporal segregation between beta and alpha suppression. Because both signals have a transient, burst-like profile—short-lived rather than sustained decreases in power—we focused on the ERD onset as the latency measure. Onset is preferable to peak here because it is less sensitive to variability in imagined speech initiation and duration across participants. In a control analysis, we also estimated sensor latencies using ERD peaks instead of onsets, which resulted in greater between-subject variability (Fig. S5). Accordingly, we band-pass filtered the MEG signal, applied a Hilbert transform to extract the analytic-signal amplitude, and computed power change relative to the − 0.5–0 s baseline. Onset latencies were defined using a slope-based criterion on the baseline-normalized envelope (see Methods). The grand-average Hilbert-envelope time courses across all sensors (Fig. 3a–c) showed that beta suppression began earlier than alpha suppression at the group level. Because group averaging can spuriously exaggerate or diminish temporal separation, we next estimated each participant’s beta and alpha onset latencies and computed a within-subject temporal delay Δt = β _onset_ – α _onset_ (negative when beta precedes alpha). This produced a distribution of delays shifted below zero, with a median delay of ~ 130 ms (Fig. 3d–e). A one-tailed one-sample t-test on Δt confirmed that beta suppression systematically preceded alpha suppression (t(39) = − 8.85, *p* < 0.001). In summary, beta ERD onset consistently preceded alpha ERD onset, with a median delay of ~ 130 ms across participants.

### Temporal segregation between auditory-alpha and motor-beta suppression

**Fig. 4:**
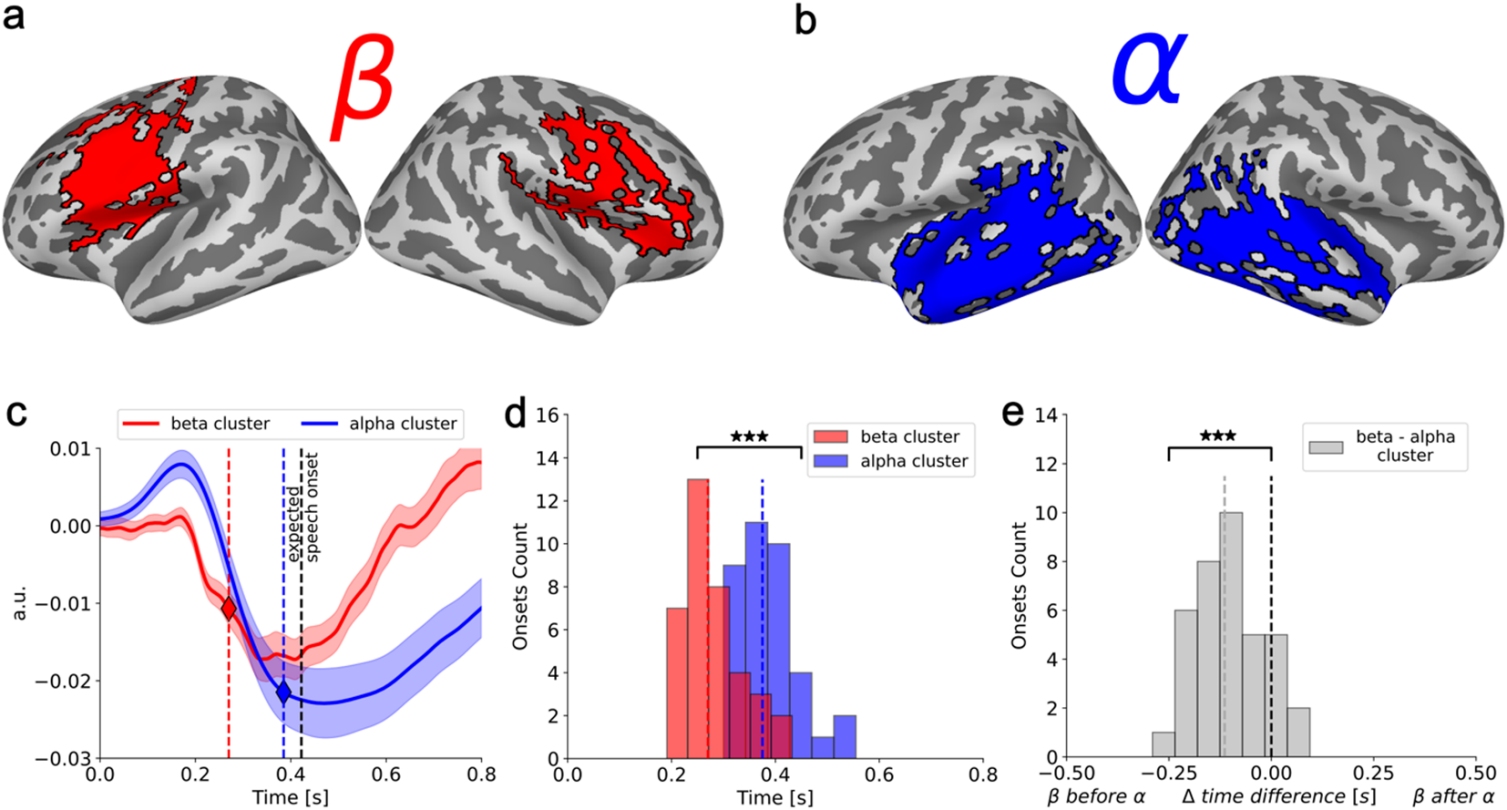
**Source space power decrease time course in beta-band frontal regions and alpha-band temporal regions**. (**a**) The red colored area delimited by black contours represents frontal regions which most prominently show the beta power decrease across participants. (**b**) The blue colored area delimited by black contours represents temporal regions which most prominently show the alpha power decrease across participants. (**c**) The average across source space vertices of the baseline normalized envelopes extracted from the frontal beta (red) and temporal alpha (blue) clusters are represented. The solid lines represent the mean and the shaded area surrounding the solid lines represent the standard error of the mean. The vertical dashed black line represents the expected speech onset measured in the behavioral pretest. The colored stars and vertical dashed lines represent the smallest frontal beta (red) and temporal alpha (blue) ERD onsets. (**d**) The distribution of individual subjects’ frontal beta and temporal alpha ERD onsets are represented in the red and blue histograms, respectively. The colored vertical dashed lines represent the median frontal beta and temporal alpha ERD onsets across participants. (**e**) The distribution of individual subjects’ temporal difference between frontal beta and temporal alpha ERD onsets is represented in the grey histogram. The black vertical dashed line represents no temporal difference; the grey vertical dashed line represents the median temporal difference between frontal beta and temporal alpha ERD onsets. A negative value indicates that the frontal beta onset precedes the temporal alpha onset, a positive value indicates that the frontal beta onset follows the temporal alpha onset.

To link sensor-space spectrotemporal dynamics to specific cortical generators, we repeated the ERD onset-latency analysis in source space, extracting beta- and alpha-band baseline normalized envelopes from functionally relevant motor and auditory regions, respectively (Fig. 4a–b). This analysis integrates the spatial information from Fig. 2 with the temporal information from Fig. 3, allowing us to test whether the observed delay reflects a motor-to-auditory sequence. We band-pass filtered the MEG signal, applied a Hilbert transform to obtain the analytic signal, projected it into source space, and computed its amplitude and power change relative to the − 0.5–0 s baseline. From these source estimates, we extracted beta activity from the motor cluster and alpha activity from the auditory cluster identified in the spatial analysis. The averaged time courses across cortical vertices in these regions (Fig. 4c) showed that motor beta suppression began earlier than auditory alpha suppression at the group level. We then estimated ERD onset latencies for each participant’s motor beta and auditory alpha suppression; onsets were defined using a slope-based criterion on the baseline-normalized envelope (see Methods). Participants who did not show a detectable beta or alpha ERD onset were excluded from this analysis (*n* = 3). In the remaining dataset, the median delay between motor beta ERD onset and auditory alpha ERD onset was approximately 120 ms (Fig. 4d–e). A one-tailed one-sample t-test confirmed that motor beta suppression systematically preceded auditory alpha suppression (t(36) = − 5.17, *p* < 0.001). In a control analysis, we measured ERD peak instead of ERD onset latencies in the motor and auditory clusters, which yielded greater between-subject variability (Fig. S6). In a second control analysis, we repeated the procedure using motor and auditory ROIs defined from a brain-atlas parcellation rather than from the data-driven spatial clusters and obtained similar results (Fig. S7), indicating that the observed temporal delay is driven primarily by activity in canonical motor and auditory regions despite the modest spatial spread of the data-driven clusters into adjacent areas. Together, these findings closely mirror the sensor-space analysis and demonstrate that the temporal delay between beta and alpha power suppression localizes to motor and auditory cortical regions.

### Correlation of alpha–beta peak latency during imagined speech with latency during overt speech

**Fig. 5:**
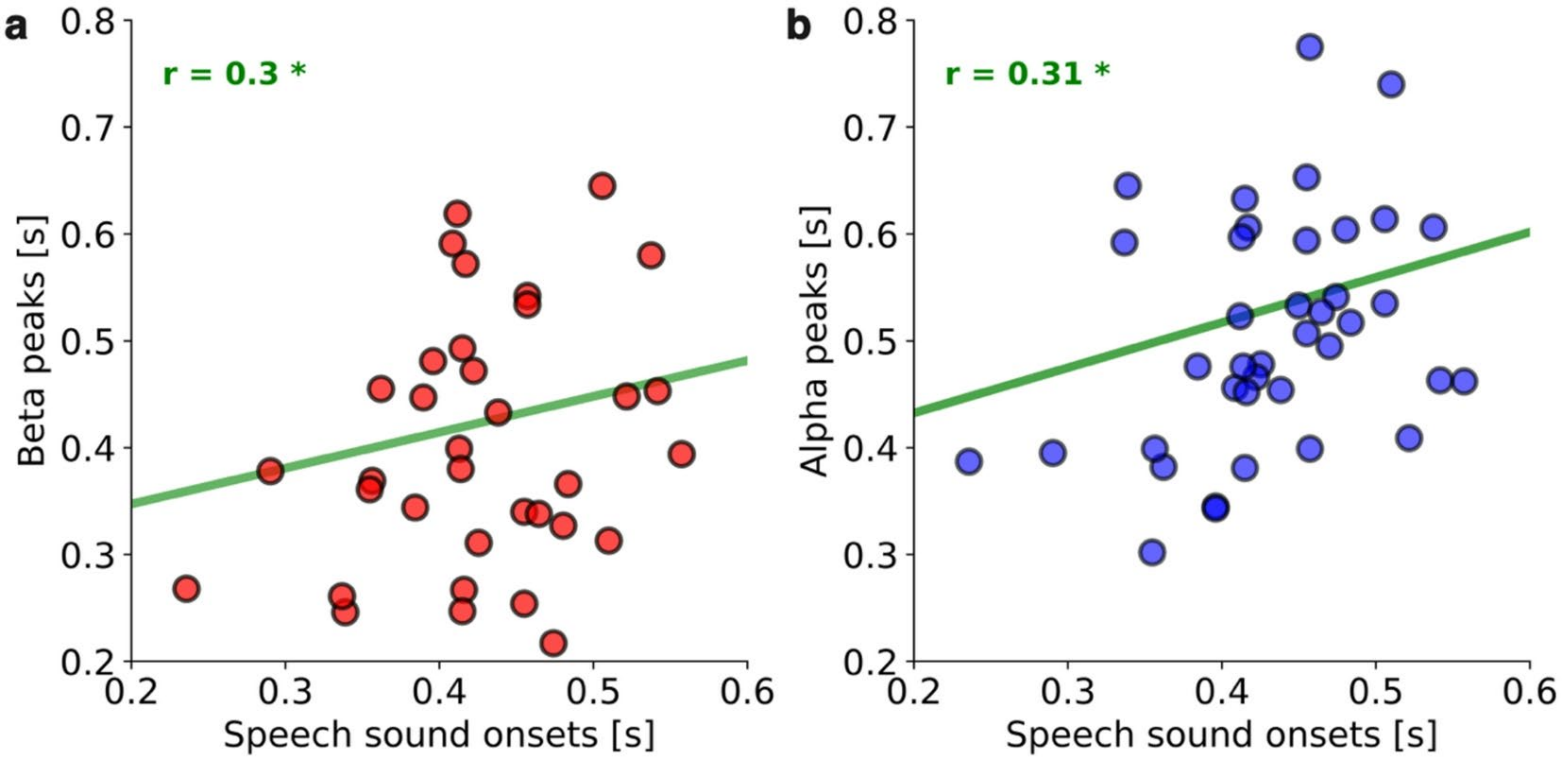
**Correlation between covert speech neural responses and overt speech behavioral responses**. A correlation between (**a**) beta (red) and (**b**) alpha (blue) power decrease peak latencies and speech production latencies as measured in the behavioral pretest is represented. Each circle represents one participant. A green regression line is shown in each panel to visualize the correlation across participants.

In the previous sections, we used ERD onset as a latency measure to reduce variability related to imagery initiation and duration across participants. At the group level, the timing of mu-rhythm power suppression during imagery shows a clear correspondence with overt speech production latency, both in sensor space (Fig. 3c) and source space (Fig. 4c). Specifically, the median beta ERD onset aligns with the micromovement onset, suggesting that beta suppression indexes the initiation of imagined articulation. By contrast, the median alpha ERD onset aligns with the speech-sound onset measured in the behavioral pre-test, suggesting that alpha suppression indexes imagined sound generation. Building on this observation, we asked whether these neural markers also track articulation timing at the individual level. Here, however, our goal was to assess whether alpha and beta power suppression timing tracks individual differences in articulation speed, so we used ERD peak latency, which better preserves this between-subject variability. Each participant has a characteristic speed for initiating speech movements, and if power suppression reflects sensorimotor coordination, then its latency should align with that participant’s speech production latency. Because articulation timing cannot be measured directly during imagined speech, we used individual speech sound onsets from the overt speech behavioral pretest as a proxy, under the assumption that imagined and overt speech share a similar temporal structure. We then tested whether alpha and beta suppression timing tracked individual differences in articulation speed. To this end, we correlated each participant’s median speech sound onset with ERD peak latencies in the motor beta and auditory alpha clusters identified in the spatial analysis and assessed significance using a permutation test (Fig. 5a–b). . The results revealed significant correlations for both motor beta (r(36) = 0.30, *p* < 0.05) and auditory alpha (r(36) = 0.31, *p* < 0.05). Importantly, these correlations were not significant when ERD peaks were estimated from all sensors, indicating that spatial localization to motor and auditory regions is critical. Together with the onset-based latency analyses reported above, these findings suggest that the timing of alpha and beta suppression is closely linked to articulation speed: beta dynamics align with the initiation of covert articulation, and alpha dynamics align with the timing of imagined sound.

## Discussion

We investigated whether the timing of sensorimotor coordination during imagined speech preserves the temporal structure observed in overt speech. Using magnetoencephalography, we identified a spatiotemporal sequence of neural events associated with imagined speech: a suppression of beta-band power in motor regions, followed by a suppression of alpha-band power in auditory regions. This sequence was consistent across participants, with motor beta suppression consistently preceding auditory alpha suppression by a delay of approximately ~ 120 ms. This time window is remarkably consistent with the sensorimotor coordination time window previously reported in overt speech studies that used auditory perturbation. This delay likely reflects a sequence of operations required to align motor and auditory information across distinct representational formats. This finding suggests that, under certain conditions, imagined speech is not temporally compressed but rather a faithful neural reenactment of the temporal scaffolding of speaking, with the key difference being the inhibition of muscle activity^21^.

### The role of stimulus complexity in temporal equivalence

We deliberately chose consonant–vowel syllabic stimuli (/pa/, /ta/, /ka/) that primarily engage low-level articulatory and auditory representations. This design allowed us to isolate and compare motor and auditory components of imagined speech more directly, thereby maximizing the chances of observing temporal equivalence with overt speech. Consistent with this approach, earlier studies investigating the rate of imagined speech using simple stimuli such as number sets or the alphabet have also reported close correspondence between overt and imagined production rates^22^. By contrast, studies using more complex stimuli such as words or sentences—which additionally recruit abstract lexical–semantic and syntactic representations—have reported temporal compression effects^23^, where imagined speech unfolds faster than its overt counterpart. Far from being contradictory, these findings are complementary: motoric and abstract linguistic representations likely co-exist during natural connected speech, with their relative dominance shaped by factors such as task demands and stimulus complexity. Our results show that, under conditions emphasizing low-level sensorimotor aspects, the timing of imagined speech closely mirrors that of overt speech.

### Beta–alpha frequency modulations as a neural marker of sensorimotor coordination

The alternation of frequency modulations that we observed align with a well-established literature on mu-rhythm event-related desynchronization (ERD). Beta suppression in motor cortex is a marker of both overt and covert movement execution^24–26^, while alpha suppression in auditory cortex is typically linked to increased excitability and enhanced processing of auditory input^27–29^. When these frequency modulations occur together in a consistent temporal sequence, they form a plausible neural signature of sensorimotor coordination. This interpretation is strengthened by the fact that the peak latencies of both beta and alpha suppression correlated with each participant’s overt speech production latencies estimated from the behavioral pre-test. The alignment with individual speech onsets suggests that both oscillatory patterns are tied to the internally simulated act of speaking, rather than reflecting unrelated processes. This is important because alpha suppression can also arise from unspecific processes, irrelevant to the task, such as attentional control^30^. If attention was the primary source of the alpha suppression, then its timing would not be expected to track participant’s speech production latency—yet our results show exactly such a correspondence. This finding makes a strong case that the observed beta–alpha suppression sequence indeed reflects sensorimotor coordination associated with imagined speech.

### Imagined speech as a tool for isolating internal motor–to-auditory progression

Across different motor control domains, overt movement execution reflects a mixture of top-down and bottom-up mechanisms. Top-down signals include motor commands and associated sensory predictions, whereas bottom-up signals arise from the physical consequences of movement, such as proprioceptive, tactile, and auditory feedback. During overt actions, these components are tightly intertwined, making it difficult to isolate the internal motor-to-sensory progression. In contrast, movements are suppressed and external sensory consequences are absent during imagery, allowing us to probe top-down mechanisms more directly. In line with this, a clear alpha–beta dissociation within the mu rhythm has been observed during the mental simulation of goal-directed grasping movements^31^, where motor imagery enhances functional interpretability by avoiding execution-related somatosensory reafference that differentially shapes post-movement alpha- and beta-band dynamics. When signal processing methods are applied to separate movement-related artifacts from neural signals—such as EMG informed artifact correction—alpha- and beta-band power suppression can be disentangled even during overt speech^32^. However, when alpha- and beta-band suppression measured during overt and covert speech are compared, suppression is consistently larger during overt speech production^33,34^, likely because concurrent articulation and sensory feedback introduce bottom-up signals that mix with top-down signals and obscure their distinct contributions. Our results show that imagined speech naturally yields a robust spatiotemporal alpha–beta dissociation that is not confounded by external sensory input and does not require artifact correction. This clear separation demonstrates that speech imagery is a powerful experimental tool for isolating sensorimotor coordination from the influence of peripheral feedback.

### Competing mechanistic accounts: direct vs. indirect mapping

The neural mechanisms underlying sensorimotor coordination remain a matter of debate. One influential proposal is that motor and auditory systems share a “common coding” scheme^35^—a ready-made, bidirectional mapping that directly translates movements into sounds and vice versa. In this direct mapping view, the link is so well entrenched that it could operate almost instantaneously. Early theoretical formulations, such as the motor theory of speech perception^36^, proposed that speech perception engages concurrent motor representations associated with (intended) speech production. Although this theory received only limited empirical support, it regained attention when neuroimaging studies reported activation of motor regions during speech perception^37^, consistent with interdependent sensorimotor representations^38^. If this account were correct, the timing of motor–auditory crosstalk should roughly correspond to a single synaptic transfer—on the order of 5–20 ms for cortical communication, depending on distance^39^. In contrast, an indirect mapping view proposes that sensorimotor coordination is mediated by an intermediate hub that transforms motor into sensory codes and vice versa. Within the framework of dual-stream models of language processing^40^, a strong candidate for this role is the Sylvian–parietal–temporal area (Spt), which is active during both speech perception and production^41^. This account predicts a longer temporal delay, reflecting at least two synaptic steps. In overt speech, such comparisons are confounded by the additional delay introduced by information traveling from brain to periphery. Instead, in imagined speech sensorimotor coordination occurs entirely within the brain, allowing a more direct assessment of the relevant time window. Time-resolved functional connectivity studies investigating coupling between motor and auditory areas during imagined speech^42^, together with our present findings, point to delays substantially exceeding those expected from a single synaptic transfer (i.e., ~ 120 ms) consistent with the involvement of an intermediate hub.

### Imagined speech as a window into the intrinsic timing of sensorimotor coordination

By combining MEG with a speech imagery paradigm emphasizing low-level acoustic and motor aspects, we uncovered an orderly spatiotemporal sequence: motor beta suppression followed by auditory alpha suppression. These frequency modulations were separated by a temporal delay closely matching the time window for sensorimotor coordination in overt speech. Crucially, imagined speech enables measurement of this temporal delay in isolation—unaffected by the byproducts of articulation. In this way, our results reveal the intrinsic duration of the sensorimotor coordination, unfolding with the same temporal scaffolding as articulation but without the confounds of overt movement execution and external feedback. The spatiotemporal dissociation between motor and auditory frequency modulations thus offers both a methodological tool and a theoretical lens for probing the time window in which the brain integrates action and perception during speech, whether imagined or spoken.

## Methods

### Participants

40 right-handed participants (mean age 27.18, range 20–47, 12 male) took part in this experiment. All participants reported no history of psychiatric, neurological, or language disorders. Moreover, participants reported normal hearing, and normal or corrected-to-normal vision. Participants’ hand preference was assessed using the Edinburgh Handedness Inventory^43^. Informed written consent was obtained from each participant in accordance with the Declaration of Helsinki. Ethical approval to conduct the study was provided by New York University Institutional Review Board (IRB).

### Stimuli

We used a set of three consonant-vowel (CV) syllables: /pa/, /ta/ and /ka/. For each syllable, we varied the consonant while we kept the vowel constant. The syllables were similar in duration and overall acoustics but distinct in motor space. Following the international phonetic alphabet (IPA) classification, the starting phonemes are unvoiced stop consonants which have different places of articulation. /p/, /t/, and /k/ are bilabial, alveolar, and velar consonants that recruit articulators in the front, mid, and back of the upper vocal tract, respectively.

### Experimental design

We tested participants in a behavioral and a neuroimaging task. The behavioral task took place right before the neuroimaging task, such that participants’ performance in the two tasks was as similar as possible. In fact, the behavioral task was designed to acquire temporal landmarks that were subsequently used as a reference to interpret neuroimaging data. This task was also aimed to establish an exclusion criterion for the neuroimaging task. This preselection was necessary to reduce the temporal variability in speech production latency across participants that is detrimental for neural data analyses.

The experimental procedure was structured as follows. There was a variable baseline period in which a fixation cross was displayed on the screen for 1–1.5 s. Then, participants were visually presented with one consonant-vowel syllable (/pa/, /ta/ or /ka/) for 1 s and instructed to imagine producing them as quickly as possible without generating overt movement or sound. After that, there was a 2.5 s inter trial interval in which a fixation cross was displayed on the screen. Participants were instructed to keep their eyes open and to maintain eye fixation during visual cue presentation. We deliberately used the visual modality, rather than auditory presentation, so that the internally generated speech representations could be isolated from stimulus-driven auditory responses. This choice forces participants to map the orthographic input onto corresponding motor and auditory output, while avoiding the stimulus–response overlap that would occur with auditory presentation as well as potential working memory confounds. To maintain consistent initiation timing across trials, syllable presentation order was pseudorandomized: the three syllables appeared in varying orders across trials but always in balanced triplets, preventing participants from anticipating or pre-activating upcoming items.

The difference between the behavioral and the neuroimaging task was that participants were instructed to speak overtly and covertly, respectively. In the behavioral task, participants spoke the syllable aloud. Participants completed one block, that is 120 trials in total. In the neuroimaging task, participants imagined speaking the syllable, without moving and without producing sounds. Imagined speech was described to participants as the internal simulation of the movements and sounds associated with speech production. Participants completed 4 blocks. Each block consisted of 120 trials; therefore, participants performed 480 trials in total. In both the behavioral and the neuroimaging task we used PsychoPy toolbox^44^ version 2, Python release 3.8.3, for stimulus delivery.

### Behavioral data acquisition and analysis

Participants were seated in front of a computer screen in a soundproof booth. We used a 16-channel, 8-preamp, 24-bit/96 kHz, MOTU system for audio input/output. Participants’ utterances were recorded using a microphone. We estimated the speech sound onsets for each participant and for each trial. As a first screening, we used a custom MATLAB script to compute the envelope of the speech sound waveform and for each trial we automatically extracted the most prominent rising of the peak that was estimated as an abrupt change in the derivative of the speech sound waveform envelope. These automatically extracted speech sound onsets measures were used to obtain an approximate estimate of participants’ temporal precision in the task. We instructed participants to be as consistent as possible across trials in terms of speech production timing. If participants’ speech onsets distribution was too broad and/or the median of the distribution was skewed towards the beginning or the end of the trial, we asked them to practice more until they improved their temporal precision. Participants who were still unable to meet the minimum requisites were excluded and did not take part in the MEG study. Specifically, we excluded participants who did not meet the following criteria: a median speech onset between 200 and 600 ms and an interquartile range smaller than 400 ms. Thirteen participants were excluded based on their performance in the behavioral pre-test and therefore were not included among the 40 MEG datasets analyzed in this study.

The speech sounds collected during the behavioral pre-test were subsequently used to annotate speech onsets for each participant and for each trial. To do that, we used the Praat software^45^. Speech sound onsets were determined by visual inspection of the spectrogram based on the characteristic signatures of unvoiced stop consonants. Unvoiced stop consonants are associated with occlusions in the vocal tract which result in specific acoustic signatures corresponding to a silent period (closure) and a noise burst (release). In particular, /p/, /t/, and /k/ show clear differences in the shape of the noise burst: /p/ shows a short-lived wide range burst across all the spectrogram that has lower intensity; /t/ shows a more prolonged burst in the upper part of the spectrogram that has higher intensity; /k/ shows an even longer burst in the lower part of the spectrogram that also has higher intensity^46^.

### EMG data acquisition and pre-processing

Participants were instructed to avoid jaw and lips movements during covert speech. Articulatory movements were continuously monitored using electromyography (EMG). We recorded the EMG signal from four electrodes: one reference electrode placed on the right mastoid, one ground electrode placed on the right wrist, one electrode placed below the cheekbone to record jaw movements and one electrode placed between the lower lip and the chin to record lips movements. Previous studies have shown that muscle movements measured from jaw and lip are sufficient to distinguish unvoiced stop consonants^47^. Electrode impedance was kept below 25 kΩ.

EMG electrodes were connected to an MEG-compatible BrainAmp DC amplifier (Brain Products GmbH, Gilching, Germany). The EMG was recorded at a sample rate of 500 Hz. A 60 Hz notch filter was applied to remove power line noise. Data was referenced online to the right mastoid. Data was re-referenced offline using bipolar derivations (jaw minus mastoid electrode; lip minus mastoid electrode) to enhance local muscle activity. A zero-phase, two-pass Butterworth bandpass filter with a 1 Hz high-pass frequency cut-off and a 50 Hz lowpass frequency cut-off was applied. We segmented the raw signal into epochs between − 1 s before 1 s after the visual presentation of the syllable. Time series were down-sampled to 250 Hz and the EMG signal was zero-meaned and detrended. A baseline correction was applied by computing the mean of the 1s baseline period preceding syllable cue onset and subtracting this mean from the entire trial epoch. We used an auto-reject algorithm for automatic artifact rejection. This algorithm defines a threshold for artifact rejection that is specific for each participant based on a cross-validation procedure. This individualized rejection threshold was motivated by the high variability across subjects in the signal-to-noise ratio of EMG recordings caused by individual differences in skin conductance, magnitude of muscle artifacts, and heartbeat artifacts.

### MEG data acquisition

Individual head shapes and fiducial landmarks (nasion, right and left pre-auricular points), were digitized using a 3D laser scan hardware (Polhemus, FastSCAN COBRA 3D) and a 3D digitizer software (Source Signal Imaging, Inc.EMSE Locator 2.0 https://cortechsolutions.com/products/emse/locator/). Five Head Position Indicator (HPI) coils were placed on participant’s mastoid bones and forehead to keep track of participant’s head position inside the dewar through electromagnetic induction. We measured head position before and after each recording block. When the maximum difference between head positions before and after each block was above 1 cm, data was excluded from further analysis. Prior to data acquisition, all metal and other potential sources of electromagnetic interference were removed. Prior to running the experiment, we recorded 3 min of MEG data without participant in the scanner (empty room recording).

MEG recordings were obtained in a magnetically shielded room (Vacuum Schmelze, Hanau, Germany) using a 157-channel whole-head axial gradiometer system (KIT, Kanazawa Institute of Technology, Japan) and 3 orthogonally oriented reference magnetometers. Participants performed the task in a supine position. When positioning participants in the MEG scanner, we ensured tight contact with the dewar. Participants were instructed to avoid head, body, and limb movements during the recording. The MEG signal was sampled at 1 kHz. Two filters were applied during data acquisition: a zero-phase two-pass Butterworth band-pass filter 1–200 Hz and a notch filter at 60 Hz. Visual stimuli were presented using a CP-X8150 LCD projector (Hitachi America LTD). Images were projected on a first-surface mirror (Edmund Scientific, Barrington, NJ) suspended from the ceiling and fixed at a 45° angle; the incoming image hit the mirror and was reflected 90° straight down. A Cedrus StimTracker was used to keep track of trigger delivery with high temporal precision.

### MEG preprocessing

MEG pre-processing was applied using MNE-Python^48^ (v0.20.7), Python release 3.8.3, combined with custom routines. First, we removed external and internal sources of noise from the recorded MEG signal. External noise (e.g., stationary noise, environmental noise) was removed offline from the MEG recordings using two denoising algorithms run in sequence. First, we used a Continuously Adjusted Least-Squares Method (CALM)^49^ using the noise recorded during the empty room. This method consists in estimating regression coefficients from reference magnetometers recorded during the empty room to regress out environmental noise from axial gradiometers recorded during the experiment. Then, we used a time shifted PCA^50^ to remove the remaining environmental noise measured by reference magnetometers during the experiment. Reference noise magnetic fields are filtered and subtracted from axial gradiometers. The filters (one per reference magnetometer/axial gradiometer pair) are obtained by delaying the reference signals, orthogonalizing them to obtain a basis, projecting the brain sensors onto the noise-derived basis, and removing the projections to obtain clean data. Internal noise (e.g., heartbeat, muscular activity, eye blinks) was reduced using Independent Component Analysis (ICA)^51^. We used a fixed-point algorithm to estimate 30 independent components on epoched data. Up to 10 components were excluded based on visual inspection of spatial topographies and latent sources (e.g., sharp, transient deflections, slow drifts, and rhythmic fluctuations).

### MEG source reconstruction

T1-weighted anatomical scans were acquired for 14 participants. When the anatomical scans were not available, we used a template average brain to perform source reconstruction^52^. The original shape of the template average brain was adjusted - either increased or decreased along x, y, z coordinates - to match participants’ head shape that was measured using the 3D laser scanner. To perform group-level analyses in a common reference frame, we computed a linear interpolation (i.e., morphing) between the decimated individual source model and a template average brain. The anatomical scans were 3D reconstructed using the Freesurferversion 7.4.1 https://surfer.nmr.mgh.harvard.edu/software^53,54^. A Boundary Element Model (BEM) was estimated using the watershed algorithm. MRI and MEG coordinate systems were co-registered by matching digitized anatomical fiducial landmarks to participant’s T1 anatomical scan. The resulting whole brain 3D mesh (5124 vertices; 6.2 mm average source spacing), the BEM model and the aligned coordinate frames were used to compute the forward model for source reconstruction. The forward model predicts the spatiotemporal characteristics of the MEG signals given a certain distribution of neural activity, considering both the conductivity properties of the head tissues and the geometry of the MEG sensor array.

The inverse problem consists in finding the optimal mapping from a low dimensional sensor space (157 axial gradiometers) to a high dimensional source space (5124 vertices). The inverse model predicts the most likely source space configuration that would produce the observed magnetic fields. The inverse solution consists in finding a set of sensor weights for each source. Weights are estimated as a combination of the forward model and inverse model. The combined forward and inverse models are optimized iteratively to minimize the mismatch between the predicted and observed MEG signals. Weights are based on the sensor locations with respect to the brain. Thus, weights are fixed and do not change over time or over frequency. We used the dSPM (distributed Source Probability Model) method for source reconstruction. The projection in source space was obtained by multiplying the data matrix with the weighting matrix. We implemented source reconstruction using MNE-Python^48^ (v0.20.7), Python release 3.8.3.

### ROI definition and extraction

We a priori selected motor and auditory regions of interest (ROIs) in the peri-Sylvian language network. We used a cortical parcellation scheme obtained using a brain atlas. The FreeSurfer software can be used to label the cortical surface into anatomical regions^55^. This procedure consists in automatically assigning a neuroanatomical label to each location on a cortical surface based on probabilistic information estimated from a manually labeled dataset. The result is a labeling of cortical sulci and gyri. We selected 2 ROIs: one motor ROI including cortical vertices anterior to the precentral gyrus, and one auditory ROI including cortical vertices located in Heschl’s gyrus, superior temporal gyrus and superior temporal sulcus regions.

For each ROI, we extracted a time course that summarizes the activity of underlying vertices. MEG measurements are insensitive to the direction of the current flow along the source. Thus, the estimated orientation of the sources is ambiguous. Source reconstruction techniques (e.g., MNE, dSPM) yield two possible orientations for each reconstructed source, corresponding to two opposite directions. We used the mean flip method for ROI extraction which ensures that the average orientation of the reconstructed sources aligns with the expected orientation based on anatomical or functional considerations. This is typically achieved by flipping the orientation of individual sources if necessary, so that their combined effect results in a consistent and interpretable orientation pattern.

### Time-frequency analysis

By definition, time-frequency decomposition methods cannot provide precise time and frequency estimates at the same time. Higher temporal resolution can only be obtained at the expense of lower frequency resolution, and vice versa. Here, we wanted to investigate both temporal and frequency aspects in detail. Therefore, we used two different time-frequency decomposition methods that are designed to optimally capture frequency and temporal aspects, respectively.

First, we used the Stockwell (S-) transform^56^ that is designed to balance temporal and spectral resolution by adjusting one single parameter. This method uses a windowed Fourier transform with a Gaussian window whose width varies with frequency. We can control the tradeoff between spectral and temporal resolution by specifying different widths of the Gaussian window. This allows for variable time-frequency resolution. Given a time-domain signal x(t), the Stockwell (S-) transform X(t, f) is calculated as follows:

1 $$\:X\left(t,\:f\right)=\:\underset{-\infty\:}{\overset{\infty\:}{\int\:}}x\left(\tau\:\right)\:\:g\left(t-\tau\:\right)\:\:{e}^{-2\pi\:if\tau\:}\:d\tau\:$$

where g(t) is a Gaussian window function centered at t. In the MNE-Python^41^ implementation the width parameter can be set to be < 1 or > 1. Where < 1 means higher temporal resolution, and > 1 means higher frequency resolution. In this study, we set the Gaussian window width parameter to 1.2 to have higher precision in the frequency domain.

Next, we used the Hilbert transform^57^ to capture time-varying oscillatory dynamics. The Hilbert transform can be used to estimate how power and phase change over time in a specific frequency band. Before applying the Hilbert transform, the signal was bandpass filtered. We filtered the broadband signal in the alpha (8–12 Hz) and the beta (15–30 Hz) frequency bands and repeated the following steps for each frequency. A Fourier transform of the bandpass filtered signal was computed. The phase quadrature component (i.e., one-quarter-cycle, 90° or π/2) is created and added to the real-valued signal. This operation is done by rotating the complex Fourier spectrum of a real-valued signal. This is equivalent to estimating instantaneous phase over time. Then, the inverse Fourier transform is computed. The combination of the original signal and its Hilbert transform is known as the “analytic signal”. The analytic signal is a complex spectrotemporal representation of the original signal which specifies its amplitude and phase as a function of time and frequency. The envelope of the analytic signal is obtained by taking its absolute value. The Hilbert transform was implemented using MNE Python^48^.

### Source power estimation

The trade-off between time and frequency resolution requires different approaches for estimating the power of the signal used as input for source reconstruction. To address this, we employed two complementary methods: one optimized for frequency resolution and the other for temporal resolution.

First, to enhance frequency resolution (at the expense of temporal resolution), we estimated Power Spectral Density (PSD). For each epoch and sensor, we measured absolute power in specific time–frequency windows determined from the group-level statistical test on the time–frequency representation (S-transform). In the trial period, PSD was computed in the following two windows: (1) 0.25–0.55 s, 15–30 Hz; and (2) 0.35–0.8 s, 8–12 Hz. For the baseline period, the corresponding windows were: (1) − 0.5–0 s, 15–30 Hz; and (2) − 0.5–0 s, 8–12 Hz. PSD was estimated using a multitaper method with Discrete Prolate Spheroidal Sequence (DPSS) windows^58^. Multiple orthogonal tapers were applied to each MEG time series, and the resulting spectra were averaged across tapers. The multitaper bandwidth was set to 4 Hz. After PSD estimation, we applied the inverse solution by multiplying the data matrix with the weight matrix, and then computed the percentage change in power during the trial period relative to the baseline period. This yielded relative power for each epoch and vertex, which was then averaged across epochs.

Second, to enhance temporal resolution (at the expense of frequency resolution), we estimated time-varying, frequency-specific power. The signal was band-pass filtered in the alpha (8–12 Hz) and beta (15–30 Hz) ranges, and the analytic signal was obtained as the combination of the filtered signal and its Hilbert transform for each epoch and sensor. We then applied the inverse solution by multiplying the data matrix with the weight matrix. Because both band-pass filtering and the inverse solution are linear operations, they can be safely combined without overlap. In contrast, the envelope of the analytic signal, obtained by taking the absolute value, is a nonlinear transformation. Therefore, we estimated the envelope in source space for each epoch and vertex. Finally, we computed the percentage change in the trial period (0–1 s) relative to the baseline (–0.5–0 s), yielding relative power for each epoch and vertex, which was then averaged across epochs.

### ERD latency estimation

Peak latency was defined as the time of the most prominent ERD minimum within the tial window. Local minima in the baseline normalized power envelope were identified as candidate ERD events, and candidates were evaluated based on their prominence relative to the surrounding signal (i.e., depth with respect to adjacent local maxima) and their separation from neighboring minima to avoid selecting minor fluctuations. The most prominent minimum was selected as the ERD peak latency for that band and/or cortical region.

Onset latency was operationalized using a slope-based criterion to accommodate between-participant differences in ERD magnitude and baseline variability. Specifically, we computed the first temporal derivative of the baseline-normalized envelope and defined onset as the earliest time point at which the derivative became reliably negative relative to baseline. To ensure that this change reflected a genuine ERD rather than transient fluctuations, candidate onsets were additionally required to exceed a minimal decrease in power relative to baseline (typically ~ 10–20% below baseline).

### Statistical analysis

To investigate the consistency of time-frequency patterns and their source localization across participants, we ran a cluster-based permutation one sample test (CBPT)^59^. CBPT is a nonparametric statistical test consisting in two different stages: a cluster formation stage and an inferential stage. In the cluster formation stage, the unit-level statistic is computed for each sensor or source. We used a t-test unit-level statistic. We computed the unit-level test statistic 1000 times. For each iteration, the data points in the time-frequency matrix or the spatiotemporal matrix were shuffled. Then, the original t values were compared to permuted t values yielding uncorrected p-values. Spatial adjacency matrices were used to define time-frequency and sources proximity. Time-frequency or sources were selected according to an a priori defined alpha criterion (i.e., *p* < 0.05) and adjacent time-frequency or sources not exceeding this value were grouped together into clusters. Finally, we summed all the t values within each cluster. Minimum cluster size was set to 10 time-frequency and 100 vertices. In the inferential stage, the summed unit-level permutation values within each cluster were used to compute the cluster-level statistical distribution under the null hypothesis of exchangeability. We calculated the percentage of clusters for which the un-permuted cluster-level statistic was larger than the permuted cluster-level statistic. If the cluster p-value was smaller than *p* < 0.05 then we assumed that the data in the two experimental conditions were significantly different. Crucially, we do not make inferences about the population based on the time-frequency and sources based on the cluster-based permutation test^60^. We only use this test as a data driven approach to detect time-frequency a source patterns consistent across subjects that are subsequently used as a starting point for more focused spatial and temporal analyses.

## Supplementary Information

Below is the link to the electronic supplementary material.

Supplementary Material 1

## Data Availability

The datasets analyzed during the current study are available from the corresponding author on reasonable request.
