# Supplementary material for: Mu rhythm motor–auditory delay in imagined speech mirrors overt speech timing
Source: Sci Rep. 2026 Jan 28;16:6528. doi: 10.1038/s41598-026-37421-1 (PMC12909796; doi:10.1038/s41598-026-37421-1)
Supplement: Supplementary file 1 — Supplementary Material 1 [file 41598_2026_37421_MOESM1_ESM.docx]

**Supplementary Material**

*Evoked Responses in MEG and EMG*

The task elicited evoked responses in both MEG and EMG signals. The grand-average event-related field across participants is shown in Fig. S1a. As expected, early responses were observed over occipital sensors, consistent with visual encoding of the stimulus. These were followed by later deflections over temporal and frontal regions. The grand-average event-related potential from jaw and lip EMG electrodes (Fig. S1b) showed a small but consistent deflection around the expected time of covert speech production, reflecting subtle peripheral engagement during the task.


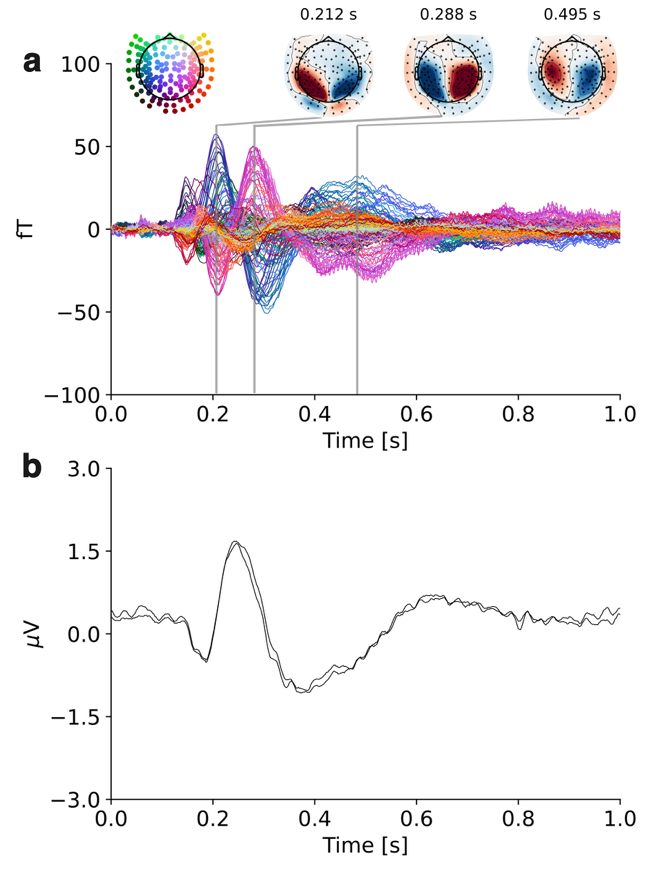


**Figure S1**. *MEG/EMG task-related evoked responses*. MEG (**a**) and EMG (**b**) grand average event-related responses. The MEG waveform consists of 157 colored lines representing the axial gradiometers. The inset topography in the upper left corner is color-coded to represent the spatial location of each sensor on the scalp. Three representative topographies illustrating the posterior to anterior progression of evoked responses are represented. The EMG waveform consists of two lines representing lip and jaw electrodes.

*Articulatory movements and micromovements impact*

A central methodological challenge in MEG studies of overt speech is the contamination by movement-related artifacts. Previous work has attempted to mitigate these artifacts using regression approaches relying on both EMG recordings and continuous head-position estimates^1^, or by applying independent component analysis seeded by EMG activity^2,3,4^. While such methods can sometimes suppress artifacts at lower frequencies, they are far less reliable at higher frequencies. Moreover, these procedures risk discarding genuine neural signals along with the artifacts. Even if movement artifacts were completely eliminated, overt speech would remain problematic: the somatosensory and auditory consequences of self-generated movements inevitably overlap with the internal motor–auditory interactions of interest. For these reasons, overt speech is unsuitable for the aims of this study. Imagined speech, by contrast, circumvents these confounds. A direct comparison of EMG recordings from overt and imagined speech (Fig. S2b,f) revealed that micromovements during imagined speech are approximately an order of magnitude smaller than overt articulations. These subthreshold involuntary movements are generally too small to overshadow the MEG signal in the same way that the muscle-movement magnetic artifact does during overt speech. Their consistent presence nevertheless indicates that participants were fully engaged in the articulation task and intentionally simulating the motor act of speaking. At the same time, these subtle deflections may reflect the task instructions, which emphasized attempted articulation rather than unconstrained inner speech. Whereas inner speech resembles a form of free thought, attempted articulation more directly recruits articulatory planning and can therefore leave a faint motor trace. Viewed in this light, micromovements are not a confound but a natural byproduct of the experimental design. The earliest prominent deflection in the grand-average EMG evoked response—corresponding to micromovement onset—occurred at approximately 250 ms (Fig. S3).


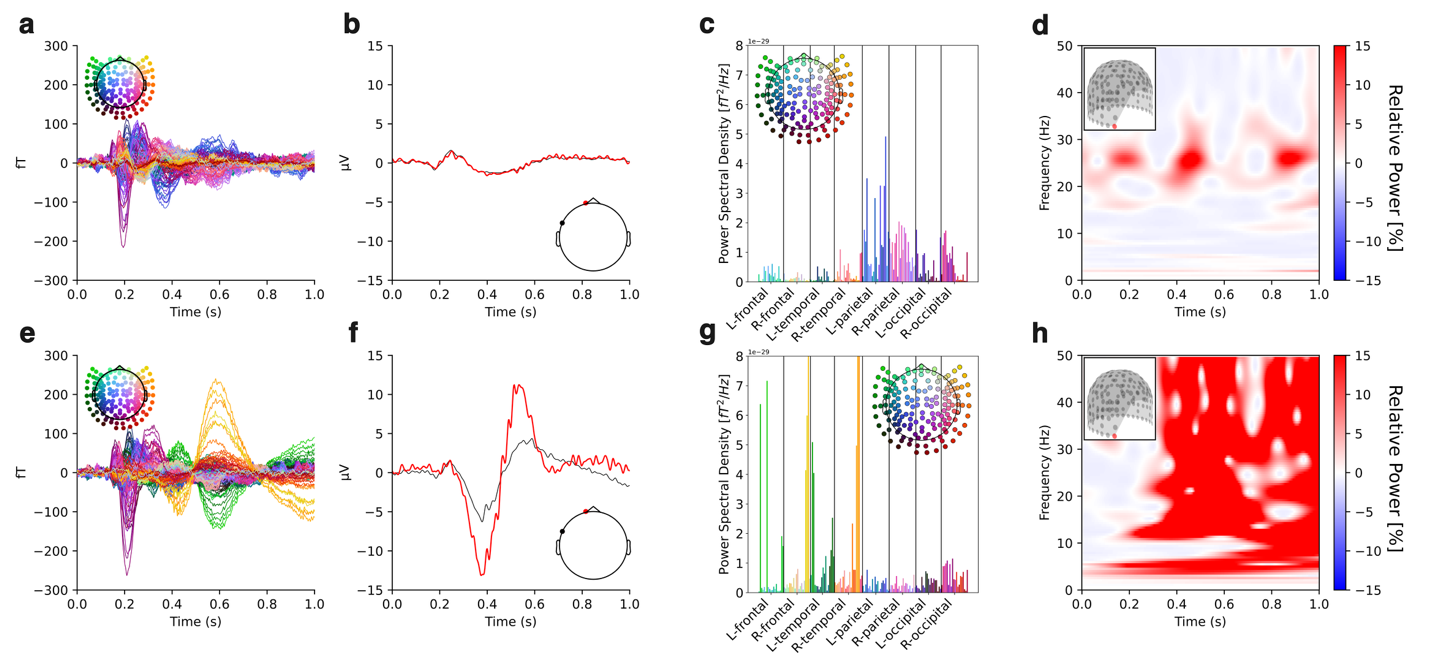


**Figure S2**. *MEG/EMG comparison between covert and overt speech*. A comparison between MEG and EMG signals measured during covert (**a**, **b**, **c**, **d**) and overt (**e**, **f**, **g**, **h**) speech production for one representative subject. Panels (**a**) and (**e**) show the event-related evoked MEG response associated with covert and overt speech production. Every line represents a different MEG sensor. MEG sensors are color-coded according to their spatial location that is shown in the topography in the upper left. Panels (**b**) and (**f**) show the event-related EMG response associated with covert and overt speech production. The red and the black lines represent the signal measured from the electrodes placed on the participant’s lip and jaw, respectively. Panels (**c**) and (**g**) show broadband power spectral density (PSD) across sensors during covert and overt speech, respectively. Each bar corresponds to a single sensor and shows the PSD estimated for that sensor. Sensors are color-coded and grouped by hemisphere (left / right) and anatomical region (frontal, temporal, parietal, occipital); the inset head-topography indicates the color-to-region mapping used for the bars. Panels (**d**) and (**h**) show the non-phase-locked time-frequency representation for one representative anterior MEG channel that is located near participants’ mouth.


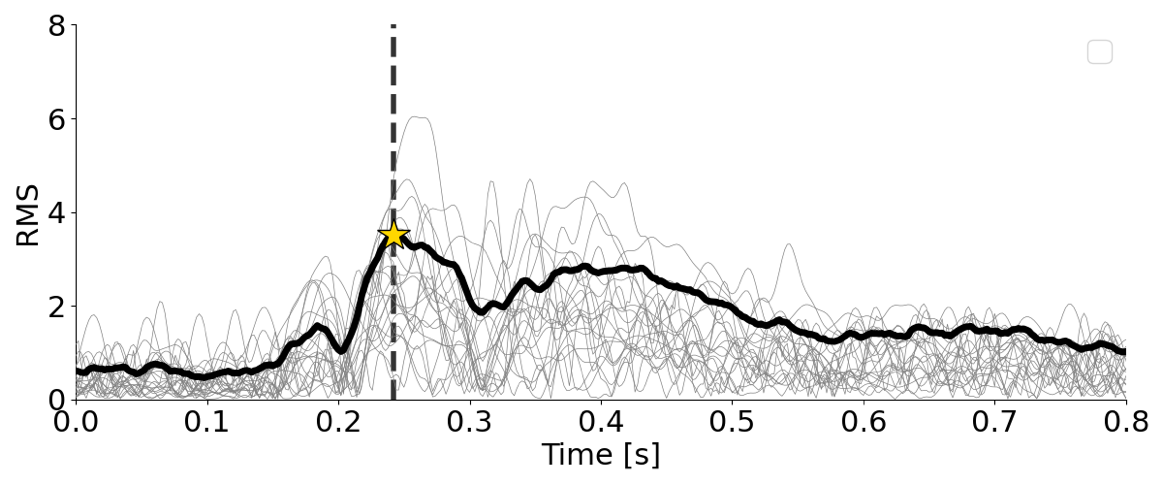


**Figure S3**. *EMG micromovements onsets*. The grand-average (N=40) EMG event-related response (thick black line) superimposed on individual participants’ event-related responses (thin grey lines). The vertical dashed line indicates the first and most prominent peak in the EMG event-related response corresponding to micromovements onset.

*Phase-locked and non-phase-locked oscillations separating evoked and induced responses*

To better characterize the relationship of power suppression with the stimulus, we explicitly separated phase-locked (evoked) and non-phase-locked (induced) frequency modulations. Biophysical modeling and electrophysiology have shown that phase-locked oscillations are typically stimulus-driven, whereas non-phase-locked oscillations reflect endogenous or self-generated processes. Following standard procedures^5^, we estimated time–frequency representations from all sensors and subtracted the evoked response (i.e., the across-trial average) from each epoch. This subtraction cancels activity that is consistently phase-aligned across trials, isolating the induced, non-phase-locked components. This analysis revealed two distinct patterns (Fig. S4a–b). First, a transient power increase in the theta band (4–7 Hz) following cue presentation was eliminated by subtracting the evoked response, indicating that it reflects stimulus-driven visual processing. Second, in contrast, the alpha (8–12 Hz) and beta (15–30 Hz) power decreases around the expected speech onset persisted after subtraction, confirming that these oscillations are non-phase-locked and therefore internally generated. These findings suggest that the alpha–beta suppression reflects internally driven brain signals, whereas the theta-band power increase originates from externally driven visual responses.


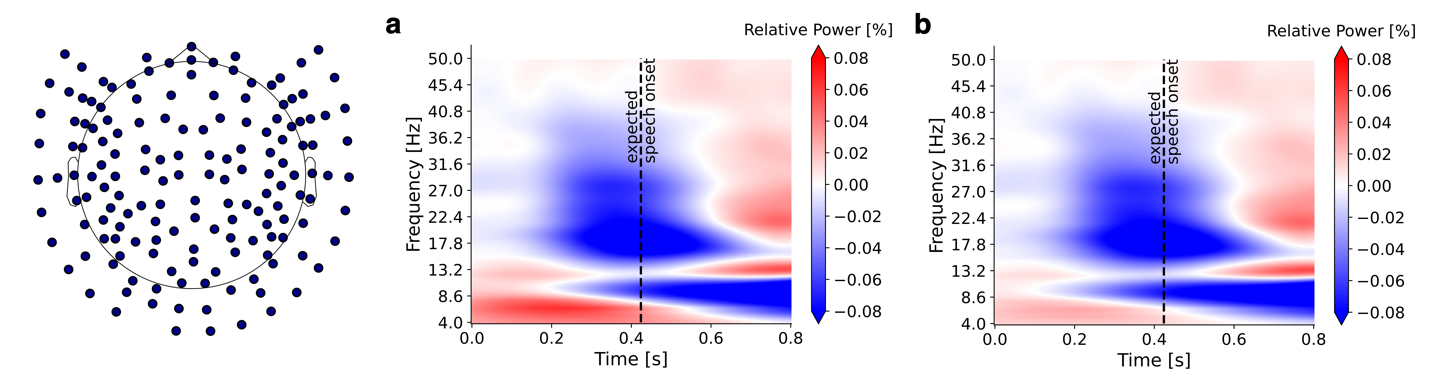


**Figure S4**. *Sensor space phase-locked and non-phase-locked time-frequency representation*. The time-frequency representation was estimated from 157 axial gradiometers. (**a**) Grand average time-frequency representation of both phase-locked and non-phase-locked power modulations. (**b**) The time-frequency representation of non-phase-locked power modulations was obtained by subtracting the average across trials from each epoch. Red and blue colors represent percentage increase and decrease in power with respect to the baseline period. The dotted vertical line represents the expected covert speech onset as estimated in the behavioral pretest.

*Peak-based alpha-beta latency definition*

To quantify the timing of alpha- and beta-band event-related desynchronization (ERD), we estimated latencies using two complementary definitions: onset and peak. Onset latencies were used for analyses focused on neural response timing because they are less sensitive to inter-subject variability associated with differences in imagined speech initiation and duration (Fig. 3-4). Peak latencies were used for individual differences analyses because they preserve this variability and therefore better capture stable between-subjects differences in the overall timing of imagined speech neural responses (Fig. 5, S5, S6, S7).


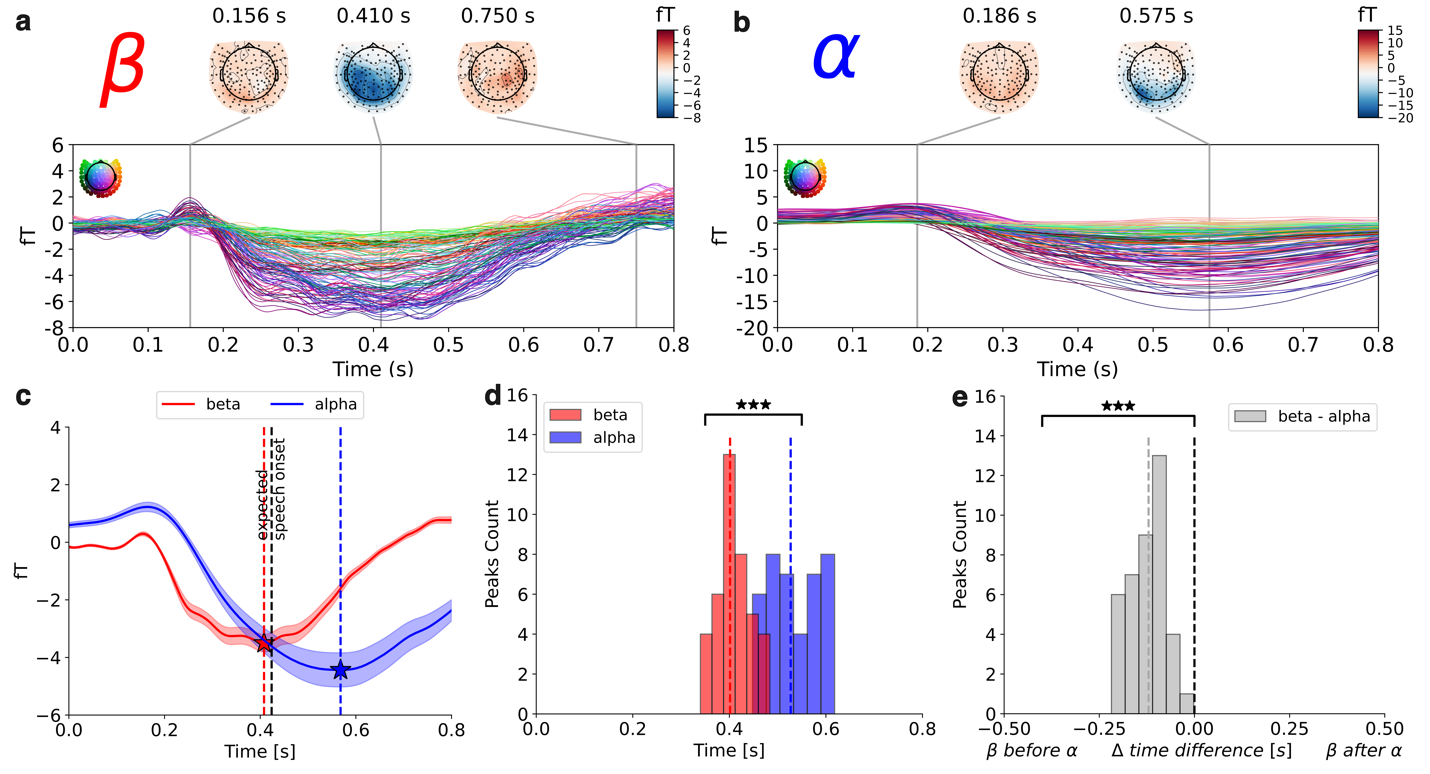


**Figure S5**. *Sensor space power decrease time course in the beta and alpha frequency bands*. Grand average Hilbert transformed band-filtered power decrease with respect to the baseline in the alpha (**a**) and beta (**b**) frequency bands. The lines represent the 157 axial gradiometers. MEG sensors are color-coded according to their spatial location on the scalp that is shown in the topography in the upper left. The topographies of most salient events in the time course are shown on top together with their timing. (**c**) The average across sensors in the beta (red) and alpha (blue) frequency band is represented. The solid lines represent the mean and the shaded area surrounding the solid lines represent the standard error of the mean. The vertical dashed black line represents the expected speech onset measured in the behavioral pretest. The colored stars and vertical dashed lines represent the smallest beta (red) and alpha (blue) peaks. (**d**) The distribution of individual subjects’ beta and alpha peaks are represented in the red and blue histograms, respectively. The colored vertical dashed lines represent the median beta and alpha peaks across participants. (**e**) The distribution of individual subjects’ temporal difference between beta and alpha peaks is represented in the grey histogram. The black vertical dashed line represents no temporal difference; the grey vertical dashed line represents the median temporal difference between beta and alpha peaks. A negative value indicates that the beta peak precedes the alpha peak, a positive value indicates that the beta peak follows the alpha peak.


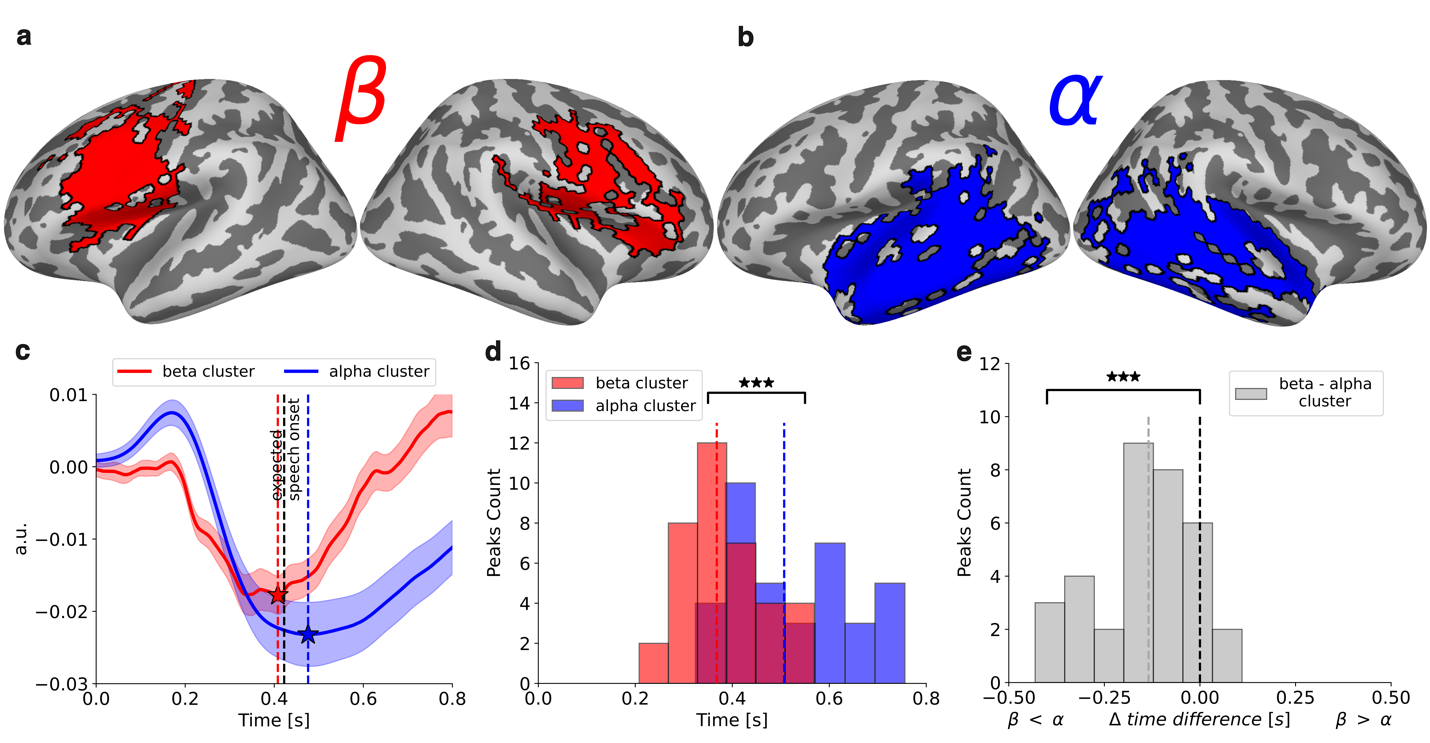


**Figure S6**. *Source space power decrease time course* *in beta-band frontal regions and alpha-band temporal regions.* (**a**) The red colored area delimited by black contours represents frontal regions which most prominently show the beta power decrease across participants. (**b**) The blue colored area delimited by black contours represents temporal regions which most prominently show the alpha power decrease across participants. (**c**) The average across source space vertices in the frontal (red) and temporal (blue) regions is represented. The solid lines represent the mean and the shaded area surrounding the solid lines represent the standard error of the mean. The vertical dashed black line represents the expected speech onset measured in the behavioral pretest. The colored stars and vertical dashed lines represent the smallest frontal beta (red) and temporal alpha (blue) peaks. (**d**) The distribution of individual subjects’ frontal beta and temporal alpha peaks are represented in the red and blue histograms, respectively. The colored vertical dashed lines represent the median frontal beta and temporal alpha peaks across participants. (**e**) The distribution of individual subjects’ temporal difference between frontal beta and temporal alpha peaks is represented in the grey histogram. The black vertical dashed line represents no temporal difference; the grey vertical dashed line represents the median temporal difference between frontal beta and temporal alpha peaks. A negative value indicates that the frontal beta peak precedes the temporal alpha peak, a positive value indicates that the frontal beta peak follows the temporal alpha peak.

*ROI-based temporal segregation between auditory-alpha and motor-beta suppression*

To validate the spatiotemporal dissociation between motor beta and auditory alpha suppression, we repeated the analysis using atlas-defined Regions of Interest (ROIs). Unlike data-driven spatial clusters, which isolate the most responsive cortical vertices, atlas-based ROIs are selected a priori and therefore inevitably include both relevant and less relevant neural populations; accordingly, this constitutes a more conservative test. ROIs were defined using a multimodal parcellation that integrates anatomical and functional criteria^6^. For the motor ROI, parcels around the central sulcus were combined, including primary motor and premotor cortex (Fig. S7a). For the auditory ROI, we included parcels covering Heschl’s gyrus, superior temporal gyrus, and superior temporal sulcus (Fig. S7b). From these ROIs, we projected the band-pass filtered, Hilbert-transformed MEG signal into source space and extracted the amplitude envelopes. The group-averaged time courses showed that beta power suppression in the motor ROI peaked earlier than alpha suppression in the auditory ROI (Fig. S7c). To assess this effect at the individual level, we detected peaks in each participant’s time course. Five participants did not show a detectable alpha and/or beta peak leaving 35 participants for analysis. In the remaining dataset, the median delay between motor beta peak and auditory alpha peak was approximately 80 ms (Fig. S7d–e). A one-tailed one-sample t-test confirmed that motor beta suppression systematically preceded auditory alpha suppression (t(34) = –3.61, p < 0.001). Taken together, these atlas-based ROI results reproduce the temporal sequence observed in the data-driven cluster analysis, albeit with reduced spatial specificity. This provides a proof of concept that the temporal segregation between motor beta and auditory alpha suppression is robust even when ROIs are defined a priori.


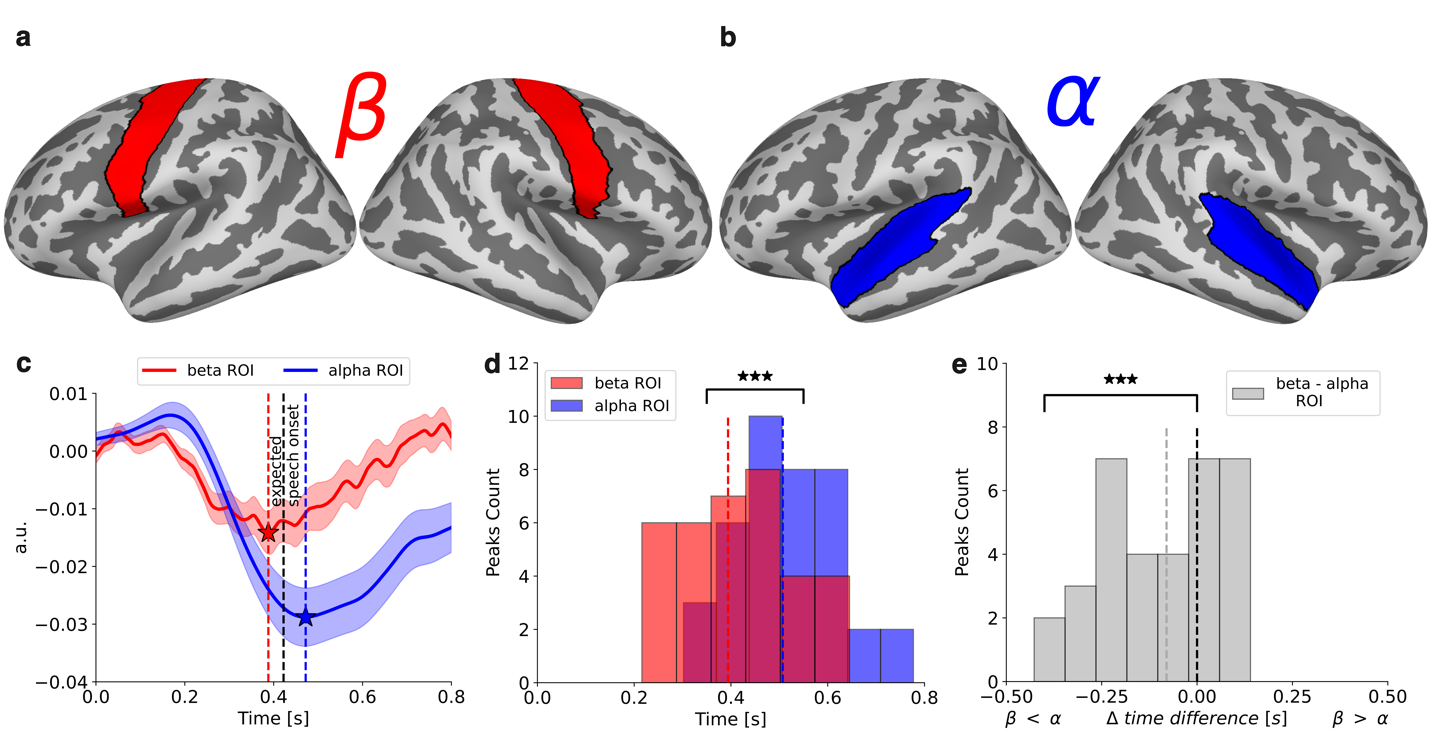


**Figure S7**. *Source space power decrease time course* *in beta-band motor ROI and alpha-band auditory ROI.* (**a**) The red colored area delimited by black contours represents the motor region of interest. (**b**) The blue colored area delimited by black contours represents auditory region of interest. (**c**) The average across source space vertices in the motor (red) and auditory (blue) ROIs is represented. The solid lines represent the mean and the shaded area surrounding the solid lines represent the standard error of the mean. The vertical dashed black line represents the expected speech onset measured in the behavioral pretest. The colored stars and vertical dashed lines represent the smallest motor beta (red) and auditory alpha (blue) peaks. (**d**) The distribution of individual subjects’ motor beta and auditory alpha peaks are represented in the red and blue histograms, respectively. The colored vertical dashed lines represent the median motor beta and auditory alpha peaks across participants. (**e**) The distribution of individual subjects’ temporal difference between motor beta and auditory alpha peaks is represented in the grey histogram. The black vertical dashed line represents no temporal difference; the grey vertical dashed line represents the median temporal difference between motor beta and auditory alpha peaks. A negative value indicates that the motor beta peak precedes the auditory alpha peak, a positive value indicates that the motor beta peak follows the auditory alpha peak.

**References**

1. Abbasi, O., Steingräber, N., & Gross, J. (2021). Correcting MEG artifacts caused by overt speech. *Frontiers in Neuroscience*, *15*, 682419.
2. Shackman, A. J., McMenamin, B. W., Slagter, H. A., Maxwell, J. S., Greischar, L. L., & Davidson, R. J. (2009). Electromyogenic artifacts and electroencephalographic inferences. *Brain topography*, *22*(1), 7-12.
3. McMenamin, B. W., Shackman, A. J., Greischar, L. L., & Davidson, R. J. (2011). Electromyogenic artifacts and electroencephalographic inferences revisited. *Neuroimage*, *54*(1), 4-9.
4. Muthukumaraswamy, S. D. (2013). High-frequency brain activity and muscle artifacts in MEG/EEG: a review and recommendations. *Frontiers in human neuroscience*, *7*, 138.
5. David, O., Kilner, J. M., & Friston, K. J. (2006). Mechanisms of evoked and induced responses in MEG/EEG. *Neuroimage*, *31*(4), 1580-1591.
6. Desikan, R. S., Ségonne, F., Fischl, B., Quinn, B. T., Dickerson, B. C., Blacker, D., ... & Killiany, R. J. (2006). An automated labeling system for subdividing the human cerebral cortex on MRI scans into gyral based regions of interest. *Neuroimage*, *31*(3), 968-980.
